# Resting state network connectivity alterations in HIV: Parallels with aging

**DOI:** 10.1002/hbm.26409

**Published:** 2023-07-07

**Authors:** Brandon J. Lew, Marie C. McCusker, Jennifer O'Neill, Sara H. Bares, Tony W. Wilson, Gaelle E. Doucet

**Affiliations:** ^1^ Institute for Human Neuroscience Boys Town National Research Hospital Omaha Nebraska USA; ^2^ College of Medicine University of Nebraska Medical Center (UNMC) Omaha Nebraska USA; ^3^ Interdepartmental Neuroscience Program Yale University School of Medicine New Haven Connecticut USA; ^4^ Department of Internal Medicine, Division of Infectious Diseases UNMC Omaha Nebraska USA; ^5^ Department of Pharmacology & Neuroscience Creighton University Omaha Nebraska USA

**Keywords:** cognitive deficits, default‐mode network, fMRI, functional connectivity, HAND

## Abstract

The increasing incidence of age‐related comorbidities in people with HIV (PWH) has led to accelerated aging theories. Functional neuroimaging research, including functional connectivity (FC) using resting‐state functional magnetic resonance imaging (rs‐fMRI), has identified neural aberrations related to HIV infection. Yet little is known about the relationship between aging and resting‐state FC in PWH. This study included 86 virally suppressed PWH and 99 demographically matched controls spanning 22–72 years old who underwent rs‐fMRI. The independent and interactive effects of HIV and aging on FC were investigated both within‐ and between‐network using a 7‐network atlas. The relationship between HIV‐related cognitive deficits and FC was also examined. We also conducted network‐based statistical analyses using a brain anatomical atlas (*n* = 512 regions) to ensure similar results across independent approaches. We found independent effects of age and HIV in between‐network FC. The age‐related increases in FC were widespread, while PWH displayed further increases above and beyond aging, particularly between‐network FC of the default‐mode and executive control networks. The results were overall similar using the regional approach. Since both HIV infection and aging are associated with independent increases in between‐network FC, HIV infection may be associated with a reorganization of the major brain networks and their functional interactions in a manner similar to aging.

## Graphical abstract

This study examined the effects of aging and HIV on resting state functional connectivity using a large sample of participants with HIV and uninfected controls age 22–72. We identify independent and additive effects of both aging and HIV on between‐network functional connectivity. This suggests HIV infection may be related to a reorganization of brain networks in a manner similar to aging.

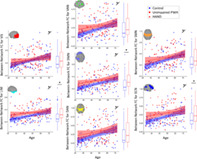

## 1 INTRODUCTION

In resource‐rich countries, people with HIV (PWH) now have a life expectancy similar to that of uninfected individuals (Samji et al., 2013). HIV eradication, however, remains elusive and HIV infection continues to be one of the leading causes of global disease burden (Ortblad et al., 2013). Additionally, with aging and long‐term chronic disease, age‐related comorbidities have become a key concern in PWH (Rasmussen et al., 2015; Wing, 2016) and studies have shown an increased incidence of such comorbidities in PWH compared with uninfected controls (Pathai et al., 2014). These comorbidities include cardiovascular disease (Martin‐Iguacel et al., 2015), liver disease (Joshi et al., 2011), renal disease (Ando et al., 2012), and neurologic diseases such as HIV‐associated neurocognitive disorder (HAND; Heaton et al., 2010). The increased prevalence of age‐related disease in PWH has led to theories of accelerated aging in PWH, which have been supported by studies of DNA methylation (Gross et al., 2016; Lew et al., 2021). Additionally, emerging literature using machine learning methods and structural MRI has also tested the hypothesis that brain aging is accelerated in PWH, finding that PWH have greater brain predicted age difference/brain age gap (Cole et al., 2017; Kuhn et al., 2018).

Studies using functional magnetic resonance imaging (fMRI) have become highly promising in identifying the changes that occur in the brain of PWH. Task‐based studies have shown patterns of hyperactivity in PWH compared with controls (Hakkers et al., 2017), including increases in prefrontal and cingulate activity during attention tasks (Chang et al., 2004, 2008, 2013) and during working memory tasks (Chang et al., 2001; Ernst et al., 2002). One meta‐analysis of such task‐based fMRI studies identified consistent HIV‐related aberrations in the fronto‐striatal circuitry (Du Plessis et al., 2014).

Similarly, a number of resting state fMRI studies have identified comparable changes in functional connectivity (FC) in PWH. That is, HIV‐related changes in FC have principally identified alterations in the frontal cortices, specifically the executive control network (ECN; Ipser et al., 2015; Ortega et al., 2015; Thomas et al., 2013) and default mode network (DMN; Cole et al., 2018; Thomas et al., 2013). Studies have also identified altered cortico‐striatal FC related to HIV infection (Ipser et al., 2015; Ortega et al., 2015). In all, these studies suggest HIV infection is related to aberrancies in frontal cortices and striatal circuits, however, the added impact of aging on these changes is not fully understood.

In uninfected individuals, normative aging appears to be related to reduced brain modularity. That is, with increasing age, within‐network FC appears to decrease, while between‐network FC increases (Betzel et al., 2014; Damoiseaux, 2017; Spreng et al., 2016). Overall, this global pattern of decreasing network segregation with aging provides a concise framework to understand how functional connectivity changes with normative aging.

Previous aging studies of HIV have been inconsistent, with some studies identifying patterns of accelerated aging in PWH as shown by HIV‐by‐age interactions (Arif et al., 2020; Chang et al., 2013; Egbert et al., 2018, 2019; Lew et al., 2020), while other studies not showing such interactions, instead finding independent and additive effects of age and HIV (Ances et al., 2010, 2012; Cole et al., 2018; Ipser et al., 2015; Juengst et al., 2007; Thomas et al., 2013). Similar inconsistencies have emerged in neuroimaging studies of HAND, with some studies finding correlations between neuropsychological performance and corresponding functional brain networks (Chaganti et al., 2017; Egbert et al., 2018; Wang et al., 2019), while others show little or no FC impairments in HAND (Cole et al., 2018; Guha et al., 2016; Janssen et al., 2017). In sum, a clear pattern of neural changes associated with HIV, aging, and cognitive function remains elusive, and few studies have examined PWH through the lens of an aging framework.

In this study, we aimed to examine the relationships between aging and HIV infection in resting state FC. Additionally, we aimed to understand the contribution of HAND toward this relationship. We studied a large sample of PWH and uninfected controls, sampled evenly from 22 to 72 years old and computed estimates of FC using resting state fMRI. To study these relationships through the lens of an aging phenotype, we assessed within‐ and between‐network connectivity, and hypothesized that PWH would show evidence of advanced aging in the form of increased between‐network connectivity, and decreased within‐network connectivity, relative to the healthy control group. We further studied how these metrics differ in participants with HAND relative to cognitively unimpaired PWH, hypothesizing that those with HAND would show larger functional aberrations and age acceleration. Lastly, to ensure the validity of our findings regardless of the brain atlas used, we conducted further analyses using regional FC.

## 2 METHODS AND MATERIALS

### 2.1 Participants

PWH were recruited from the HIV Clinic of the University of Nebraska Medical Center and demographically matched uninfected control participants were recruited from the local community. To optimize the study of aging in HIV, participants were recruited using a decade‐classification approach with manual tuning to create an even distribution across age, ranging from 22 to 72 years. All PWH were receiving effective combination antiretroviral therapy (cART) and had an undetectable viral load within 3 months of participation in the study, defined as <50 copies/mL. Uninfected controls were specifically recruited to match PWH based on their race/ethnicity, age, and sex. Of note, CD4 metrics were not examined in controls as we did not hypothesize that fluctuations in CD4 would be functionally meaningful in those without HIV. Exclusion criteria included any chronic medical illness affecting CNS function (other than HIV‐infection/HAND), any neurological or psychiatric disorder (other than HAND), acute intercurrent illness, pregnancy, history of head trauma, current substance use disorder, and presence of any ferrous metal implant which may interfere with the MRI data acquisition. The Institutional Review Board at the University of Nebraska Medical Center approved this protocol. Each participant provided written informed consent, and all participants completed the same protocol. Out of the 121 PWH and 133 uninfected controls recruited for this study, 105 PWH and 116 controls successfully completed the sMRI and fMRI protocols, and 86 PWH and 99 uninfected controls were ultimately retained after data quality exclusions (Table 1). All PWH were virally suppressed with a median current CD4 of 701.5 cells/μL (range: 102–2617) and a median CD4 nadir of 234 cells/μL (range: 3–586).

**TABLE 1:** Demographic information of the participants.

	Uninfected controls (*n* = 99)	PWH (*n* = 86)		Controls vs. PWH
		Unimpaired (*n* = 55)	HAND (*n* = 31)	
Chronological age (years; mean/SD)	45.02 (15.41)	48.80 (12.63)	46.84 (13.13)	*p* = .146
Sex (M/F; *n*/%)	55/44 (55.5/44.4%)	34/21 (61.8/38.2%)	19/12 (61.3/38.7%)	*p* = .493
Race (Caucasian, African American, Asian, Other: *n*/%)	70/22/5/2 (70.1/22.2/5.1/1.0%)	37/13/2/3 (67.3/23.6/3.6/5.5%)	18/13/0/0 (58.1/41.9/0/0%)	*p* = .437
Average composite neuropsychological *z*‐score (mean/SD)	−0.08 (0.61)	−0.09 (0.37)	−0.97 (0.41)	*p* < .001
Time since HIV diagnosis (years; mean/SD)	—	10.89 (7.25)	12.00 (7.54)	—
CD4 Nadir (median/range)	—	233.5 (3–586)	237 (7–468)	—
Current CD4 (median/range)	—	702 (102–1799)	697 (143–2617)	—

### 2.2 Neuropsychological testing

A comprehensive neuropsychological testing panel was included in the visit, including the Wide Range Achievement Test 4 (WRAT‐4), Hopkins Verbal Learning Test‐Revised (HVLT‐R), Trail Making Test (TMT) Parts A and B, Grooved Pegboard Test (GPT; dominant and nondominant hand), Verbal Fluency Test, Semantic Fluency Test, digit symbol and symbol search in the Wechsler Adult Intelligence Scale (WAIS‐III), and Stroop Test. Additionally, the Lawton‐Brody Activities of Daily Living Scale and participant's self‐assessment of own functioning, and Beck Depression Inventory were collected. For determination of HAND, the study followed the same protocol as described in Lew et al. (2018). Briefly, Composite scores for multiple functional domains (executive functioning, attention, speed of processing, fine motor, verbal learning and memory, and language) were computed by calculating demographically normalized *z*‐scores and taking an average of the *z*‐scores for all tests within that domain. Along with an assessment of activities of daily living, these scores were used to diagnose HAND according to the Frascati guidelines (Antinori et al., 2007). A global composite score was created by averaging across domains.

### 2.3 Scanning parameters

MRI data were acquired with a 3 T Philips Achieva X‐series scanner using an eight‐channel head coil. The resting‐state fMRI sequence was collected with a T2* sequence (repetition time [TR] = 2000 ms; echo time [TE] = 35 ms; voxel size = 1.7 × 1.7 × 3.5 mm; 90° flip angle; 240 mm field of view, 33 axial slices; 480 volumes). Participants were instructed to stay awake while closing their eyes. In the same session, a high‐resolution T1‐weighted anatomical scan was collected (TR = 8.09 ms; TE = 3.7 ms; 256 × 256 matrix; voxel size = 0.9375 × 0.9375 × 1 mm; sense factor: 1.5).

### 2.4 Resting‐state fMRI preprocessing

The rs‐fMRI data were preprocessed using SPM12 and the DPABI Toolbox (Yan et al., 2016). Preprocessing procedures included removal of the first 10 volumes, motion correction to the first volume with rigid‐body alignment; co‐registration between the functional scans and the anatomical T1‐weighted scan; linear detrending; regression of motion parameters, and their derivatives (24‐parameter model; Friston et al., 1996), as well as white matter (WM), cerebrospinal fluid (CSF) time series (using a component‐based noise reduction method, five principal components; Behzadi et al., 2007); and spike censoring (volumes with a framewise displacement [FD] ≥0.5 were regressed out; Satterthwaite et al., 2013); spatial normalization of the functional images into Montreal Neurological Institute (MNI) stereotaxic standard space; and spatial smoothing with a 6‐mm at full‐width at half‐maximum Gaussian kernel. Finally, bandpass filtering was applied at (0.01–0.1) Hz (Cordes et al., 2001).

For quality control of the imaging data, participants with excess head motion, defined as >0.8 in mean FD (Power et al., 2012) and/or >2.5 mm of maximum motion, were excluded from the study. After removing these individuals, 86 PWH and 99 uninfected controls had both structural and functional MRI data that could be used for further processing (Table 1).

### 2.5 Extraction of network functional connectivity measures

We used the Yeo 7‐network atlas (Yeo et al., 2011), a previously established functional brain atlas based on 1000 resting state fMRI scans, to partition the functional connectome into seven replicated resting‐state networks (RSNs; Doucet et al., 2019): visual (VIS), somato‐motor (SMN), dorsal attention (DAN), ventral attention (VAN), limbic (LIM), ECN and DMN networks. In each participant, Fisher Z‐transformed Pearson's correlation coefficients were computed to calculate FC within and between networks. Within‐network functional connectivity (WN‐FC) was computed as the average correlation of each voxel's BOLD signal time series with every other voxel within the network. Between‐network functional connectivity (BN‐FC) was computed as the correlation between the average time‐series of one network and that of all the other networks.

### 2.6 Statistics

We utilized linear regression models to examine the independent and interactive effects of HIV and aging. Each within‐ and between‐network functional connectivity metric was used as a dependent variable, HIV status was entered as a categorical independent variable, participant age was used as a continuous independent variable, and participant head motion (mean framewise displacement) was added as a covariate of no interest. An additional model was then created by adding in the HIV by age interaction, focusing on the added significance of the interaction. Additionally, 200 repeated, 10‐fold, leave‐one‐out cross‐validation was performed to further validate our within and between network models (de Rooij & Weeda, 2020). Subsequent matrix statistics also included participant head motion as a covariate of no interest, and multiple comparisons were accounted for by calculating FDR *q* values. Cells with both *p* < .05 and FDR *q* < .05 were considered significant.

### 2.7 Analysis of HIV‐associated neurocognitive disorder

To determine the impact of HAND, we performed post hoc analyses repeating our statistical comparisons after splitting the PWH group by HAND. Specifically, we performed pairwise comparisons between uninfected controls, unimpaired PWH, and participants with HAND. These comparisons were performed for all within‐ and between‐network functional connectivity estimates.

### 2.8 Parallel analyses with network based statistics

To ensure that our findings were not dependent on the technique and brain atlas used, we further conducted analyses using a regional approach. To do so, we first extracted FC between each pair of regions, based on the 512‐region automated anatomical labeling (AAL) atlas (Crossley et al., 2013; Tzourio‐Mazoyer et al., 2002; Zalesky, Fornito, Harding, et al., 2010). We then, use the network‐based statistic (NBS) toolbox v1.2 (Zalesky, Fornito, & Bullmore, 2010). NBS is a validated non‐parametric method of controlling the family‐wise error rate (FWER) when performing mass univariate testing on all connections of a network. We computed the *t*‐score for each pairwise connection separately and then determined a primary component‐defining threshold of the *t*‐scores at *t* > 5 to identify a set of suprathreshold edges. Since NBS results are highly dependent on this primary‐defining threshold, we tested additional NBS analyses with thresholds flanking the primary one (*t* = 4.5 and *t* = 5.5). Note that although the choice of this component‐defining threshold affects the sensitivity of the method, the control of FWER is guaranteed regardless of threshold choice (Zalesky, Fornito, & Bullmore, 2010). To compare the functional matrices of the two groups, permutation testing (5000 permutations) was then applied to determine to significance of each component. Components with a corrected *p* < .05 were considered statistically significant. We used this threshold to identify suprathreshold links and the size (extent) of the connected components. NBS was performed to identify sets of connections that exhibited greater connectivity in the PWH compared with the control, and vice versa, and to determine the effect of age. All NBS analyses were conducted with mean head motion as a covariate of no interest.

## 3 RESULTS

### 3.1 Effects of aging or HIV in within‐network functional connectivity

**FIGURE 1:**
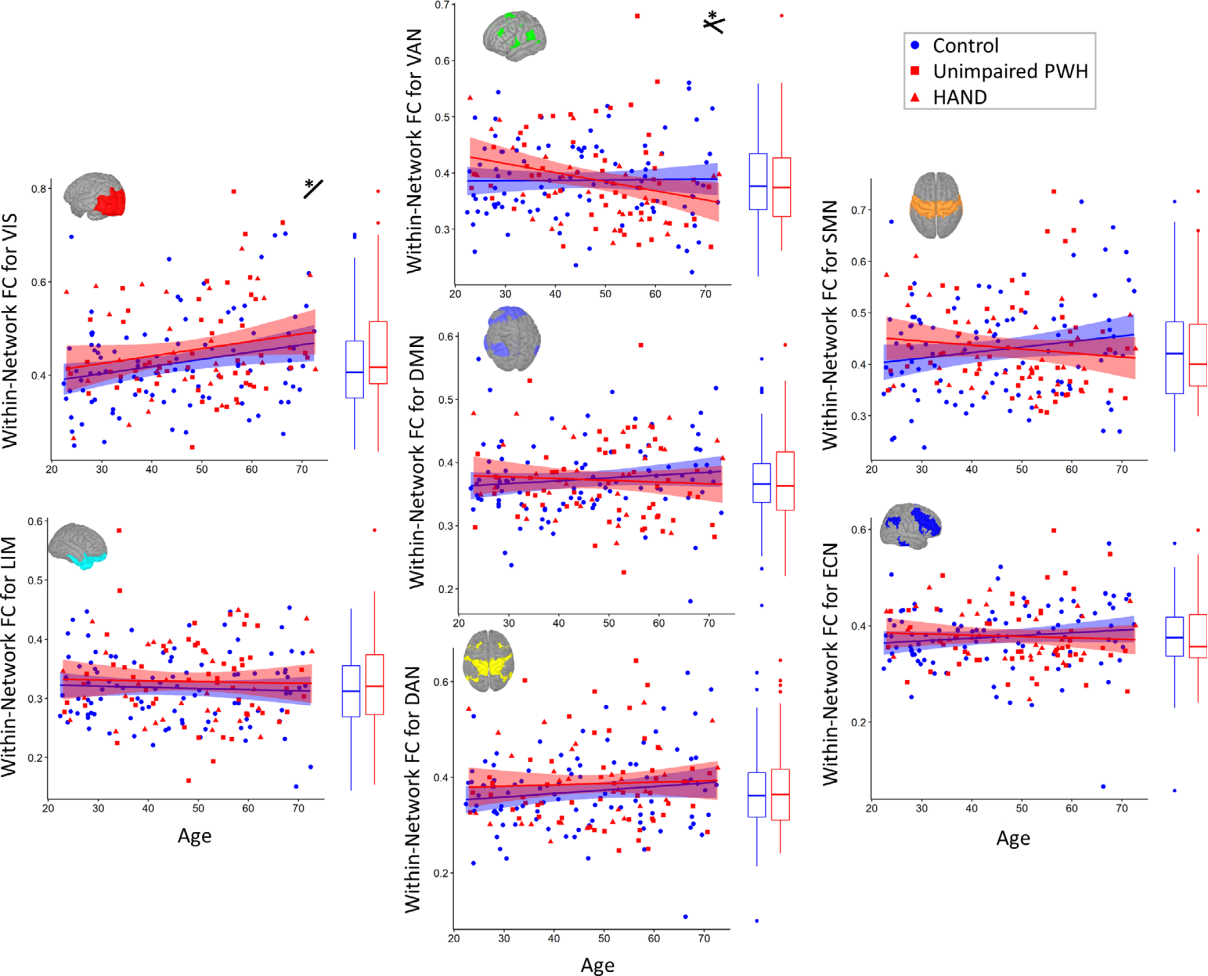
Within‐network functional connectivity by age and HIV status. The average correlation within each network was calculated and *z* transformed. Regressions testing the independent effects of HIV and age showed no significant main effects of HIV, and a significant main effect of age only in the visual network. Scatter plots display *z* values of each within‐network functional connectivity metric by age, with uninfected controls in blue and PWH in red. HAND status is differentiated by shape for display purposes. Linear fits for each group are displayed with 95% confidence intervals. Boxplots displaying group differences are added to the right of each plot, and visual representations of each network are inset. 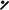 denotes significant (*p* < .05) main effect of age. 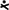
denotes significant (*p* < .05) HIV by age interaction. Related statistics are reported in Table 2.

Within‐network connectivity was estimated for seven established functional networks, and regressions were used to examine for the main effects of age and HIV. Across all seven networks, only the FC within the VIS network showed a significant main effect of age (*F*(1,182) = 9.25; *p* = .003, *ƞ* ^2^ = .047). Additionally, no main effects of HIV were identified in any of the seven WN‐FC metrics (all *p* > .05; Table 2 and Figure 1).

**TABLE 2:** Average network statistics.

Within‐network FC															
	Model 1 (no interaction term)						Model 2 (interaction term included)								
	Age			HIV			Age			HIV			HIV × age		
	*F*(1,181)	*p*	*η* ^2^	*F*(1,181)	*p*	*η* ^2^	*F*(1,180)	*p*	*η* ^2^	*F*(1,180)	*p*	*η* ^2^	*F*(1,180)	*p*	*η* ^2^
VIS	4.88	.028*	0.05	1.48	.225	0.02	3.04	.083	0.05	0.09	.770	0.02	0.00	.955	0.00
SMN	0.05	.831	0.00	0.02	.895	0.00	1.6	.208	0.00	2.99	.085	0.00	3.40	.067	0.02
DAN	0.5	.481	0.01	0.89	.348	0.01	0.73	.395	0.01	0.54	.465	0.01	0.23	.629	0.00
VAN	3.03	.083	0.01	0.00	.959	0.00	0.02	.887	0.01	4.35	.039*	0.00	4.66	.032*	0.03
LIM	1.22	.271	0.00	0.78	.379	0.01	0.91	.341	0.00	0.02	.886	0.01	0.01	.911	0.00
ECN	0.43	.512	0.00	0.00	.983	0.00	1.47	.226	0.00	1.24	.266	0.00	1.33	.249	0.01
DMN	0.46	.500	0.00	0.01	.908	0.00	1.42	.234	0.00	1.03	.310	0.00	1.20	.275	0.01

Between‐network FC															
	Age			HIV			Age			HIV			HIV × age		
	*F*(1,181)	*p*	*η* ^2^	*F*(1,181)	*p*	*η* ^2^	*F*(1,180)	*p*	*η* ^2^	*F*(1,180)	*p*	*η* ^2^	*F*(1,180)	*p*	*η* ^2^
VIS	8.11	.005*	0.08	0.14	.705	0.01	7.90	.005*	0.08	0.89	.347	0.01	0.76	.384	0.00
SMN	11.48	<.001*	0.13	2.09	.150	0.04	11.45	<.001*	0.13	2.14	.145	0.04	1.20	.274	0.01
DAN	7.64	.006*	0.09	2.61	.108	0.04	13.85	.007*	0.09	1.61	.206	0.04	0.71	.400	0.00
VAN	7.36	.007*	0.10	2.93	.089	0.05	7.96	.005*	0.10	2.29	.132	0.05	1.14	.286	0.01
LIM	7.30	.008*	0.09	5.82	.017*	0.06	8.42	.004*	0.09	3.45	.065	0.06	1.48	.225	0.01
ECN	7.38	.007*	0.10	4.89	.028*	0.06	7.79	.006*	0.10	2.57	.110	0.06	1.03	.312	0.01
DMN	9.8	.002*	0.11	5.52	.020*	0.07	8.64	.003*	0.11	1.82	.179	0.07	0.50	.480	0.00

*Note*: Two models are reported, one without the HIV by Age interaction term (Model 1), and one with the interaction term included (Model 2). Wherever the HIV by Age interaction is not significant, the main effects from model 1 are interpreted. To further correct for motion, both models include head motion as a covariate of no interest.

Abbreviations: DAN, dorsal attention network; DMN, default mode network; ECN, executive control network; LIM, limbic network; SMN, somato‐motor network; VAN, ventral attention network; VIS, visual network.

* *p* < .05.

However, adding in the HIV by age interaction to the models showed that WN‐FC of the VAN had a significant HIV by age interaction such that PWH showed a greater decrease in connectivity with age relative to controls (*F*(1,181) = 4.70, *p* = .031, *ƞ* ^2^ = .025). None of the other within‐network models displayed a significant HIV by age interaction (all *p* > .05; Table 2).

### 3.2 Effects of aging and HIV in between‐network functional connectivity

**FIGURE 2:**
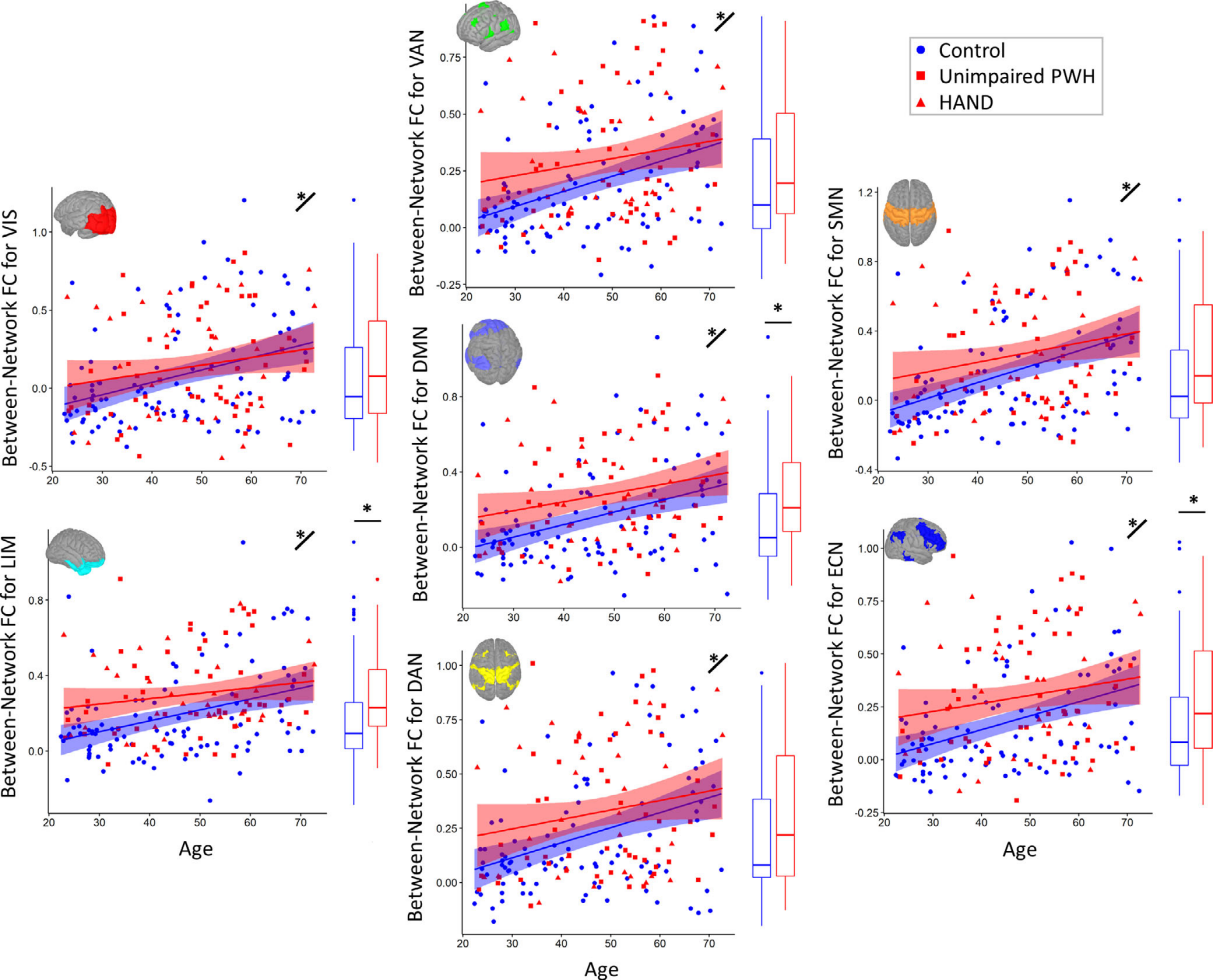
Between‐network FC by age and HIV status. The correlation between each network was calculated, *z* transformed, and then averaged for each network. Regressions testing the independent effects of HIV and age showed significant main effects of age in every network. Significant main effects of HIV were identified in limbic, default mode, and executive control networks. Scatter plots display *z* values of each between‐network functional connectivity metric by age, with uninfected controls in blue and PWH in red. HAND status is differentiated by shape for display purposes. Linear fits for the groups (controls and PWH) are displayed with 95% confidence intervals. Boxplots displaying group differences are added to the right of each plot, and visual representations of each network are inset. *p < .05 with motion covaried out. 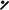 denotes significant (*p* < .05) main effect of age. Related statistics are reported in Table 2.

Significant main effects of age were identified in all seven BN‐FC metrics (see Table 2 and Figure 2). All of these effects showed consistent increases in between‐network functional connectivity with increasing age.

With regard to HIV status, BN‐FC metrics showed a significant main effect of HIV status in the LIM, DMN, and ECN (Table 2, Figure 2), All of these significant effects showed an increase in BN‐FC in PWH relative to controls. Adding in the HIV‐by‐age interaction to the models showed no interaction effects in any of the BN‐FC metrics. To further validate and explore these effects, we also performed a leave‐one‐out cross validation on all within‐ and between‐network functional connectivity models. These results supported the general findings and are reported in Figures S1 and S2.

Because of the significant impact of preprocessing on FC (Tozzi et al., 2020), we also examined these BN‐FC data after applying Global Signal Regression during preprocessing, and interrogated the impact of such processing on long versus short‐range connections. Results showed overall consistent patterns and a weak relationship between motion correlation and connectivity distance, which are reported in Figures S3 and S4).

**FIGURE 3:**
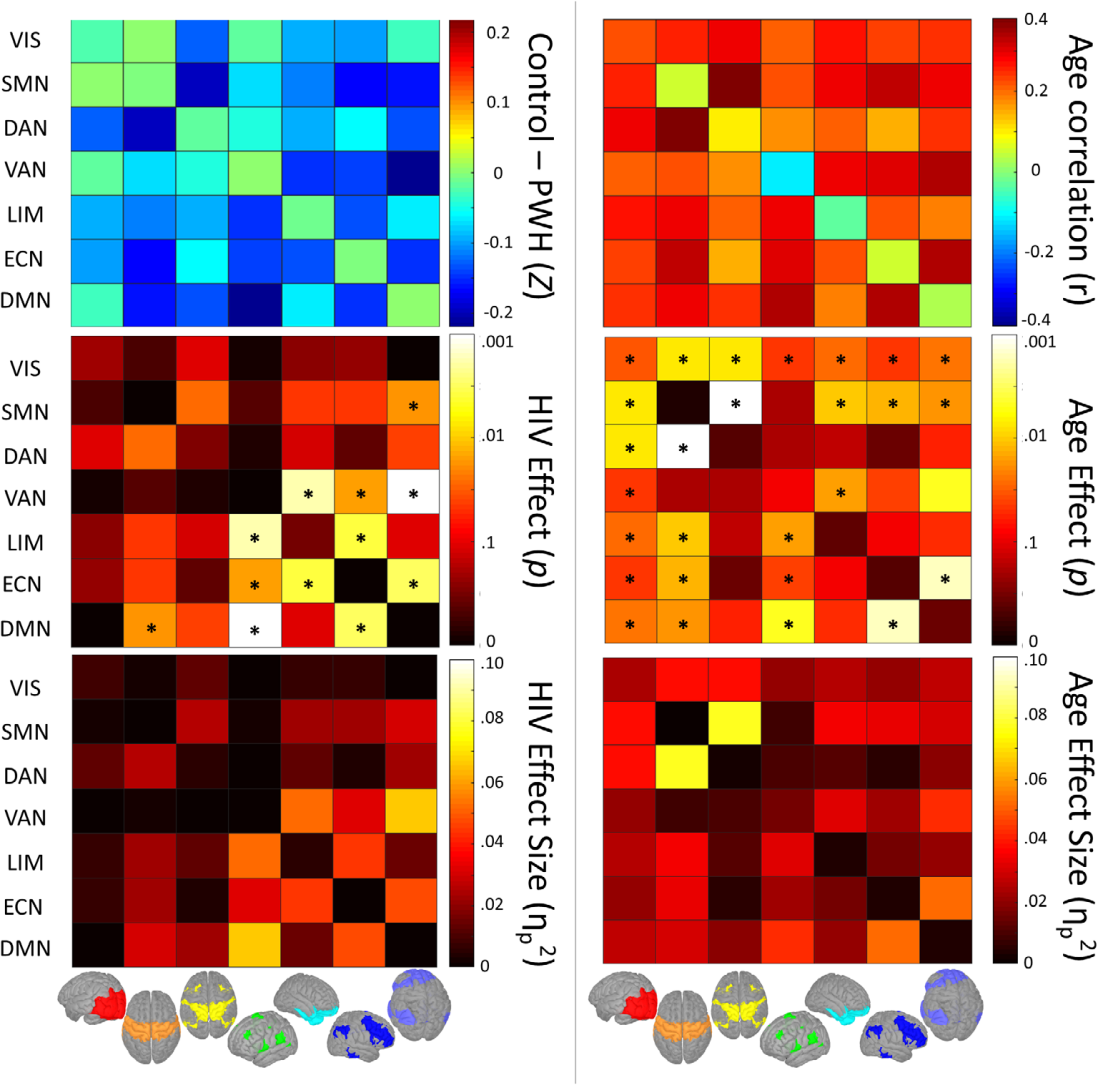
Pairwise functional connectivity statistics: effects of age and HIV. Matrix statistics performed on between‐ and within‐ (diagonal) network connectivity matrices allowed pairwise between‐network connectivity to be examined. Top left: Subtraction matrix with hot colors representing PWH < controls and cool colors representing PWH > controls. Top right: Simple age correlation matrix with hot colors representing increasing connectivity with age and cool colors representing decreasing connectivity with age. Middle: Significance matrices for the main effects of HIV (left) and age (right) displaying p values on a log scale. Asterisks denotes significant when correcting for multiple comparisons (FDR *q* < .05). Bottom: Effect size matrices for the main effects of HIV (left) and age (right) displaying *ƞ*_*p*_^2^ values. Matrices are symmetric with labels on the left, and visual representations of each network displayed on the bottom.

To further probe the effects of HIV and age on BN‐FC, we performed additional statistics on the pairwise connectivity matrices to determine whether the main effects of age and HIV effects were related to specific pairs of RSNs. After applying a multiple‐comparisons correction (FDR *q* < 0.05), the main effect of age revealed widespread significant effects, showing that the increase in BN‐FC with age was not driven by a particular set of networks (Figure 3). After correction for multiple comparisons, the most frequent main effects of HIV were detected in the FC pairs involving the VAN, ECN, and DMN with PWH showing higher BN‐FC than the control group (Figure 3). Group average and standard deviation matrices are shown in Figure S5. Additionally, matrices using the 512‐regions atlas are shown in Figure S6.

### 3.3 HAND impact on network FC

To examine whether our effects of HIV were driven by participants with HAND, we split the HIV group by HAND status and performed comparisons between controls, unimpaired PWH, and those with HAND. Ultimately, no significant differences were identified between unimpaired PWH and those with HAND. Similarly, significant differences between controls and unimpaired PWH were not identified in BN‐FC. However, significant differences between controls and PWH with HAND were present in FC between the VAN, ECN, and DMN between‐network FC metrics (all *p* < .05; Figure S7).

Given the significant group‐by‐age interaction in WN‐FC of the VAN, we also performed follow‐up pairwise comparisons of this interaction effect. The control versus HAND comparison of WN‐FC showed a significant interaction of group by age (*p* = .032), while in the control versus unimpaired PWH comparison there was not a significant group by age interaction (*p* = .143; Figure S8). This suggests that cognitive impairment may be driving this differential aging trajectory.

### 3.4 Parallel analyses with network‐based statistics

**FIGURE 4:**
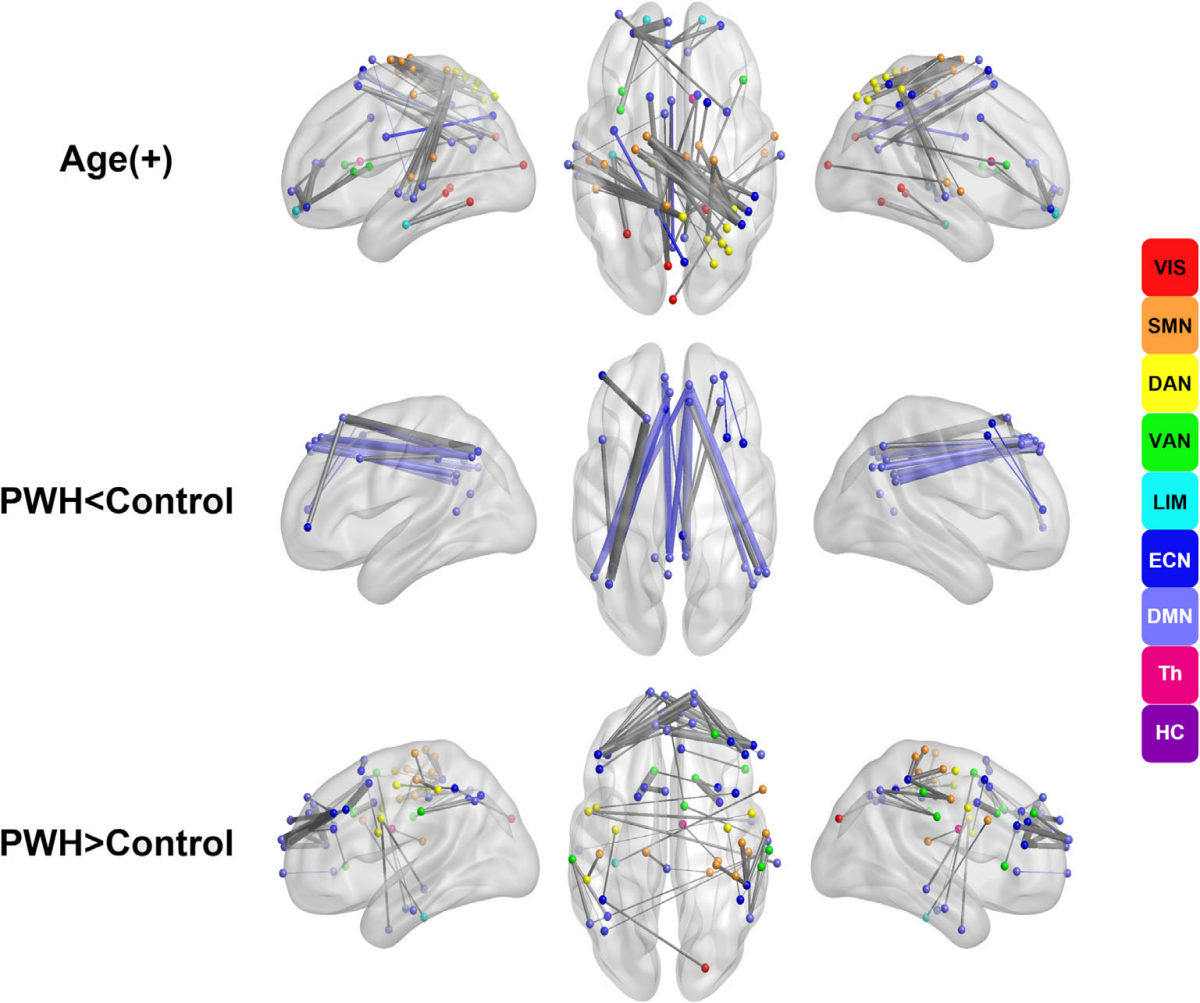
Results from the network‐based statistics toolbox. Significant functional connectivity (FC) with age and between groups with primary threshold *t*‐score = 5. (top) FC positively correlated with age, with a large majority being between‐networks throughout the brain (gray connections). (middle) Significantly weaker FC in PWH compared with uninfected controls, primarily between nodes in the frontal, parietal, and occipital regions within the DMN, within the ECN, and between the DMN and ECN. (bottom) Significantly stronger FC in PWH compared with controls, especially between nodes in the frontal regions between the DMN and ECN. Legend to the right presents the color of the nodes (and edges for within‐network connectivity) representing functional networks based on the Yeo 7‐network atlas. DAN, dorsal attention network (yellow); DMN, default mode network (periwinkle); ECN, executive control network (dark blue); HC, hippocampus (purple); LIM, limbic network (light blue); SMN, somato‐motor network (orange); Th, thalamus (pink); VAN, ventral attention network (green); VIS, visual network (red).

We conducted NBS analyses to corroborate and expand upon the findings from the network‐based FC analyses using a region‐based approach. Since the size of a significant component or subnetwork is associated with the chosen primary threshold in the NBS test, we found more significant networks for the lower threshold (*t* = 4.5) and fewer for the higher threshold (*t* = 5.5), as expected. Nonetheless, the patterns of FC were very similar. A total of 49 significant links with increasing FC with older age were identified, showing consistent results with our network‐based findings (Figure 4). The vast majority of the significant links were between nodes from different networks (gray links on Figure 4).

In regard to the similarity of the HIV effect on the network‐related findings, PWH showed significant increases in FC in a total of 24 links, which were largely between the frontal regions of the DMN and ECN (light and dark blue), as well as between the nodes belonging to the ECN and VAN (green; Figure 4). In contrast to the network approach, the NBS also identified significantly reduced FC links in the PWH compared with controls. However, these links were relatively limited and largely localized within the DMN, and particularly the dorsal part of the network (dorsal medial prefrontal cortex vs. precuneus/angular gyri; light blue, Figure 4).

Finally, no significant HIV‐by‐age interactions or differences between PWH and HAND were detected.

## 4 DISCUSSION

Our study examined a large sample of PWH and uninfected controls spanning age 22–72 and identified independent effects of age and HIV in resting‐state FC between the major brain networks. Age‐related increases in BN‐FC were widespread, and PWH displayed further increases above and beyond aging, specifically between networks related to higher order functions, such as the default‐mode and executive control networks. Employing a complementary regional‐based approach further validated these results and extended our findings by displaying the specific nodes that showed the largest effects. These findings broadly support the framework that HIV infection leads to alterations in brain functional connectivity that are highly similar to the aging phenotype. Below we discuss these findings in the context of previous literature.

Our data showed expected increases in BN‐FC with increasing age, which is a well‐established pattern of aging (Damoiseaux, 2017). Using our framework, we additionally found that HIV infection was also related to widespread increases in BN‐FC, independent and additive to the effects of aging. Our findings may be consistent with previous network level investigations of HIV, which found more positive BN‐FC in PWH compared with controls (Thomas et al., 2013). Importantly, our results expand upon these previous studies by studying this pattern across aging, establishing that this pattern occurs across adulthood and mimics the aging phenotype seen in our sample. Overall, these findings suggest that PWH display a connectivity profile that resembles an advancement in age.

These network‐level resting‐state FC findings were confirmed and expanded upon by employing the NBS toolbox (Zalesky, Fornito, Harding, et al., 2010). NBS detected altered FC at the brain region level, offering a finer granularity of the FC differences. While we found an overall similar pattern to the network‐based findings, we identified interesting results of both stronger and weaker FC in the PWH relative to the uninfected controls. Similar to the resting‐state networks analysis, NBS revealed widespread BN‐FC changes with increasing age, significant group effects where PWH had increased FC between the ECN and DMN compared with controls, no significant age‐by‐HIV interactions, and no significant main effects of HAND. In particular, there was significantly stronger FC in PWH compared with controls, especially between nodes in the frontal regions among the DMN and ECN, which has been reported in the literature (Ipser et al., 2015; Ortega et al., 2015; Thomas et al., 2013). Also, reduction in DMN FC has been reported in multiple resting‐state studies of PWH (Cole et al., 2018; Thomas et al., 2013). Here we replicate and expand upon prior studies (Ances et al., 2010, 2012; Ipser et al., 2015; Juengst et al., 2007; Thomas et al., 2013) by showing that these changes are present at the regional level, independent of aging. In fact, at the network level we did not observe lower FC within the DMN, but this did emerge at the regional level, with lower FC between the dorsal medial prefrontal cortex and angular gyri/precuneus, suggesting that HIV may specifically impact the dorsal part of the DMN over its ventral part. Taken together, our findings suggest a decrease in network modularity related to HIV infection, where PWH have weaker FC in the dorsal DMN and stronger FC between the DMN and the other higher‐order networks. Previous studies have also tended to support a decrease in modularity related to HIV (Abidin et al., 2020).

Our analyses of HAND status showed no significant differences between unimpaired PWH and HAND, suggesting our effects were not driven by cognitive dysfunction. However, when comparing these subgroups to uninfected controls, BN‐FC with the VAN, DMN, and ECN were significantly increased in those with HAND, but not unimpaired PWH. Additionally, other follow‐up analyses also suggested that participants with HAND drove the interaction effect in WN‐FC of the VAN. Caution is needed when interpreting these pairwise findings, as unimpaired PWH did not significantly differ from participants with HAND. However, taken together, this may indicate that the VAN is particularly sensitive to cognitive impairment. Supporting this, previous studies have also found HAND‐related differences in the salience network (Chaganti et al., 2017) and therefore this network may be a promising indicator of HAND. Previous studies have also identified the presence of functional deficits related to HIV in the absence of HAND, with one study specifically noting no difference in resting‐state FC by HAND status (Guha et al., 2016). This ultimately suggests that a reorganization of brain networks occurs irrespective of neuropsychological changes. At the same time, our sample of PWH with HAND may not represent a severe enough sample to detect further exacerbations of functional connectivity. That is, the majority of our participants with HAND were of the mildest category (asymptomatic neurocognitive impairment). This could have led our HAND group to be similar to the unimpaired group. Further study is needed to examine the more severe presentations of HAND and to reconcile these data with that from magnetoencephalographic (MEG) imaging, which has frequently reported neurophysiological differences among cognitively impaired (i.e., HAND) and unimpaired PWH (Arif et al., 2020; Groff et al., 2020; Lew et al., 2018; Spooner et al., 2020; Wiesman et al., 2018; Wilson et al., 2016).

Regarding HIV‐by‐age interactions, none of the BN‐FC models showed any significant interactions. This is in agreement with multiple studies showing that the effects of age and HIV on brain function are independent (Ances et al., 2010; Cole et al., 2018; Juengst et al., 2007; Ortega et al., 2015). However, importantly, these independent main effects of HIV and aging still fit into an aging framework. A significant HIV‐by‐age interaction would show that the trajectory of aging is differing with HIV infection, suggesting accelerated aging over time. Our data show coincident independent main effects of HIV and aging in many networks' BN‐FC measures, such that the trajectory of these networks remain the same, but there is an added insult related to HIV infection that is in the same direction as age‐related degradation. Thus, HIV‐related alterations in FC may be seen as an isolated advancement in aging. This model is consistent with multiple studies suggesting HIV infection causes a “hit” to biological systems, advancing the aging process (Pathai et al., 2014). One notable exception in our data is that we did identify a significant HIV‐by‐age interaction in the ventral attention network, such that PWH showed a greater decrease in FC compared with controls. Indeed, other neuroimaging studies have identified HIV by age interactive effects (Lew et al., 2021; Petersen et al., 2022), which may suggest an accelerated aging profile. Therefore, an accelerated age‐related pattern of decreased within network connectivity may have been seen in our PWH, albeit specific to one network. Interestingly, this is the same network that showed HAND‐specific changes in BN‐FC. Ultimately, the ventral attention network may be altered differentially with HIV infection, and further studies might examine this network more specifically.

Interestingly, we also identified increased WN‐FC of the VIS network with age. While it has been previously reported that this network is among the least influenced by aging (Doucet et al., 2021), other studies have also reported a positive association with aging (Seidler et al., 2015; Zhou et al., 2023; Zonneveld et al., 2019). We believe that such differences may be related to a variety of factors such as different brain atlas parcellations (Doucet et al., 2019) and sample size and diversity (Marek et al., 2022), or different preprocessing steps (Power et al., 2012). Ultimately, this network did not show any group effects and future study is needed to validate this finding.

While our study has several strengths, such as a large sample size of PWH and the use of parallel analyses involving both network and region‐based approaches, it also has a number of limitations that must be considered. Firstly, while our dataset of PWH throughout aging is relatively large given the population, much larger samples are needed to ensure replicability and address generalizability (Marek et al., 2022). While we performed a cross validation with our data, further studies using an independent dataset are needed to confirm these results are robust. Similarly, we chose a relatively lenient threshold for head motion exclusion and did not use global signal regression (GSR) in our main analyses. Regarding head motion, there is currently no clear consensus on what exact threshold should be used and it is relatively accepted that it needs to be adapted to each study/clinical sample. Accordingly, in line with previous literature (Ciric et al., 2017), we chose instead to use a censoring approach (censored volumes were regressed out) and we further used the mean head motion as a covariate in all group analyses. As per GSR, the use of GSR in preprocessing has also been a relatively controversial step and previous studies have suggested that GSR may distort group differences (Saad et al., 2012), as it might remove meaningful FC differences between clinical versus healthy groups. In this context, we focused on the main effect of age on FC and found relatively consistent results, even after GSR (Figure S1). The main difference was detected in the limbic network, with a reverse pattern in PWH. However, we believe this may be partially due to the instability of the limbic network, as shown in studies suggesting this network has relatively low reliability and high inter‐subject variability, due to its location in the lower part of the brain where the signal‐to‐noise ratio is often lower than the other brain networks (Chen et al., 2015; Sbaihat et al., 2022). Additionally, we identified a weak relationship between distance and motion correlation, suggesting long versus short range connections may be differentially impacted by motion (Ciric et al., 2017). It will be important for future studies to adopt more strict criteria for motion exclusion, as well as test the impact of GSR on resting‐state FC in an independent sample of PWH. Secondly, as mentioned previously, our sample of PWH had very well‐managed HIV‐infection in the form of effective cART and undetectable viral loads. Our sample also had no other substantial neurologic/psychiatric comorbidities, and therefore, these data may not generalize well to a broader population of PWH that may have a variety of other complications. Second, we did not examine common health comorbidities such as obesity, hypertension, or diabetes, nor did this study assess the effects of socioeconomic factors or health habits. Further study is needed to examine the impact of these other common health factors on our findings. Finally, future studies should examine older adults (beyond age 72) and PWH with more severe types of HAND.

In conclusion, HIV infection may be related to a reorganization of BN‐FC in a manner similar to aging. Specifically, both HIV infection and aging are associated with independent increases in BN‐FC. The effect of HIV was driven by increases in connectivity between default mode and executive control networks, and further examination showed that the dorsal subdivisions of the default mode network were highly affected in PWH. Additionally, these effects were identified in a sample of virally suppressed PWH and were present in those with and without HAND, suggesting such reorganization occurs despite successful viral suppression and regardless of cognitive impairment. In sum, our findings illuminate the network‐level changes that occur with HIV infection and aging, and may contribute to our understanding of the pathophysiology underlying HIV‐related neural alterations.

## AUTHOR CONTRIBUTIONS

**Brandon J. Lew:** Conceptualization, Formal analysis, Investigation, Writing – original draft, Writing – review & editing, Visualization, Supervision; **Marie C. McCusker:** Conceptualization, Formal analysis, Writing – original draft, Writing – review & editing, Visualization; **Jennifer O'Neill:** Methodology, Supervision; **Sara H. Bares:** Methodology, Supervision; **Tony W. Wilson:** Conceptualization, Writing – review & editing, Visualization, Supervision, Funding acquisition; **Gaelle E. Doucet:** Conceptualization, Writing – review & editing, Visualization, Supervision.

## FUNDING INFORMATION

Supported by the National Institutes of Health (NIH; MH103220, MH116782, MH062261, DA041917, DA048713, DA047828, DA056223, GM144641, AG064001) and the National Science Foundation of the USA (NSF; 1539067).

## Supporting information


**Data S1.** Supporting Information.

## Data Availability

The data that support the findings of this study are available from the corresponding author upon reasonable request.
