# Supplementary material for: Resting state network connectivity alterations in HIV: Parallels with aging
Source: Hum Brain Mapp. 2023 Jul 7;44(13):4679–91. doi: 10.1002/hbm.26409 (PMC10400792; doi:10.1002/hbm.26409)
Supplement: Supplementary file 1 — Data S1. Supporting Information. [file HBM-44-4679-s001.docx]

**Resting State Network Connectivity Alterations in HIV: Parallels with Aging**

Authors: Brandon J. Lew, Marie C. McCusker, Jennifer O’Neill, Sara H. Bares, Tony W. Wilson, Gaelle E. Doucet

**Supplementary Material**


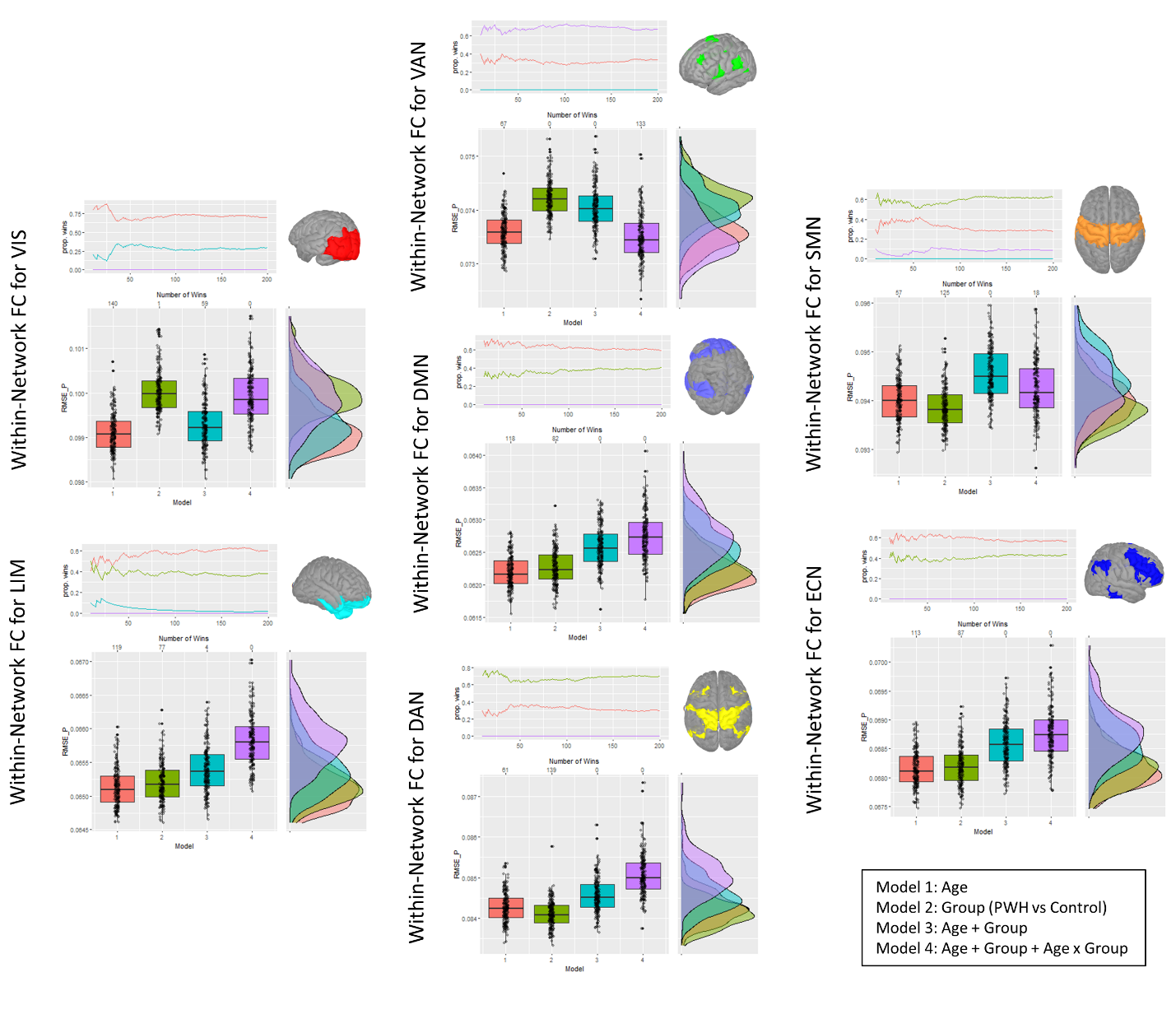


*Figure S1: Within-Network Functional Connectivity Leave-One-Out Cross Validation: We performed 200 repeated, 10-fold, cross-validation on each of our within network models (de Rooij et al. 2020). Each cross-validation compared the root mean squared error (RMSE) of four models predicting connectivity of the respective network: 1. age, 2. group (HIV status), 3. age + group, and 4. age + group + age*group. Effectively, this compares the model performance for the addition of each respective term, with model 3 containing the two independent effects, and model 4 containing the interaction. For each network, the upper panel displays the proportion of wins for each model with each repetition number. The panel below displays boxplots of the four models’ prediction error (root mean square error of prediction, RMSEp); each box indicates the middle 50% of values, with the horizontal line inside the box indicating the median, and the vertical lines representing the range from lowest to highest prediction error, excluding outliers. The number of wins for each model is shown at the top. The panel to the right of the boxplot is a graph of the density estimates for the prediction errors of the competing models. Finally, a graphic representation of each network is inset on the top right. Overall, models including only the single effect of age (model 1) had the lowest error in a majority of within-network models. Only the ventral attention network (VAN) had the interaction model out-perform lower order models.*


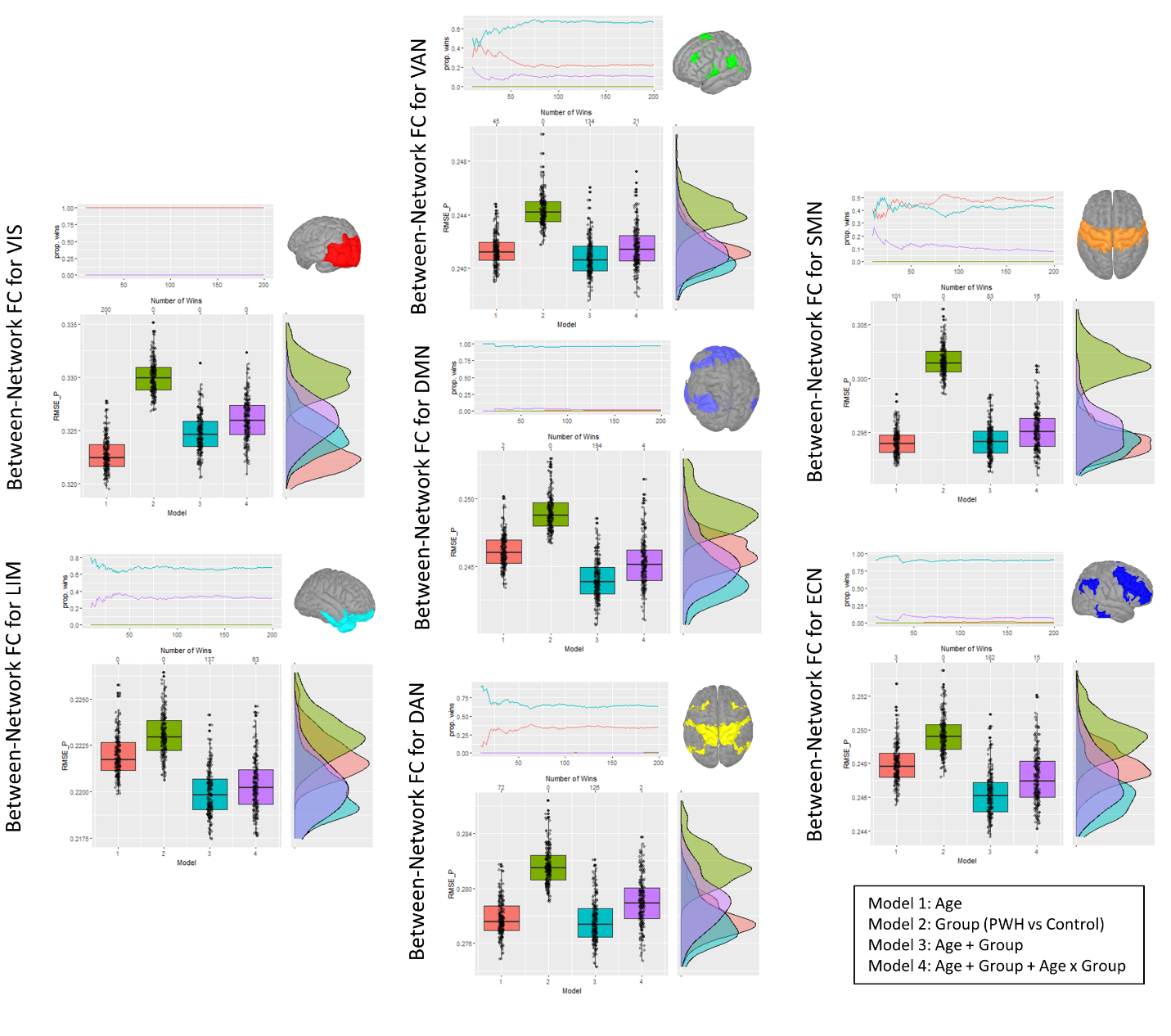


*Figure S2: Between-Network Functional Connectivity Leave-One-Out Cross Validation: We performed 200 repeated, 10-fold, cross-validation on each of our between network models (de Rooij et al. 2020). Figure layout is as presented in Figure S1, with lower RMSE indicating better model performance. Overall, models including the independent effects both of age and HIV status (model 3) had the lowest error in a majority of between-network models. This supports that age and HIV have independent and additive effects on between-network functional connectivity.*


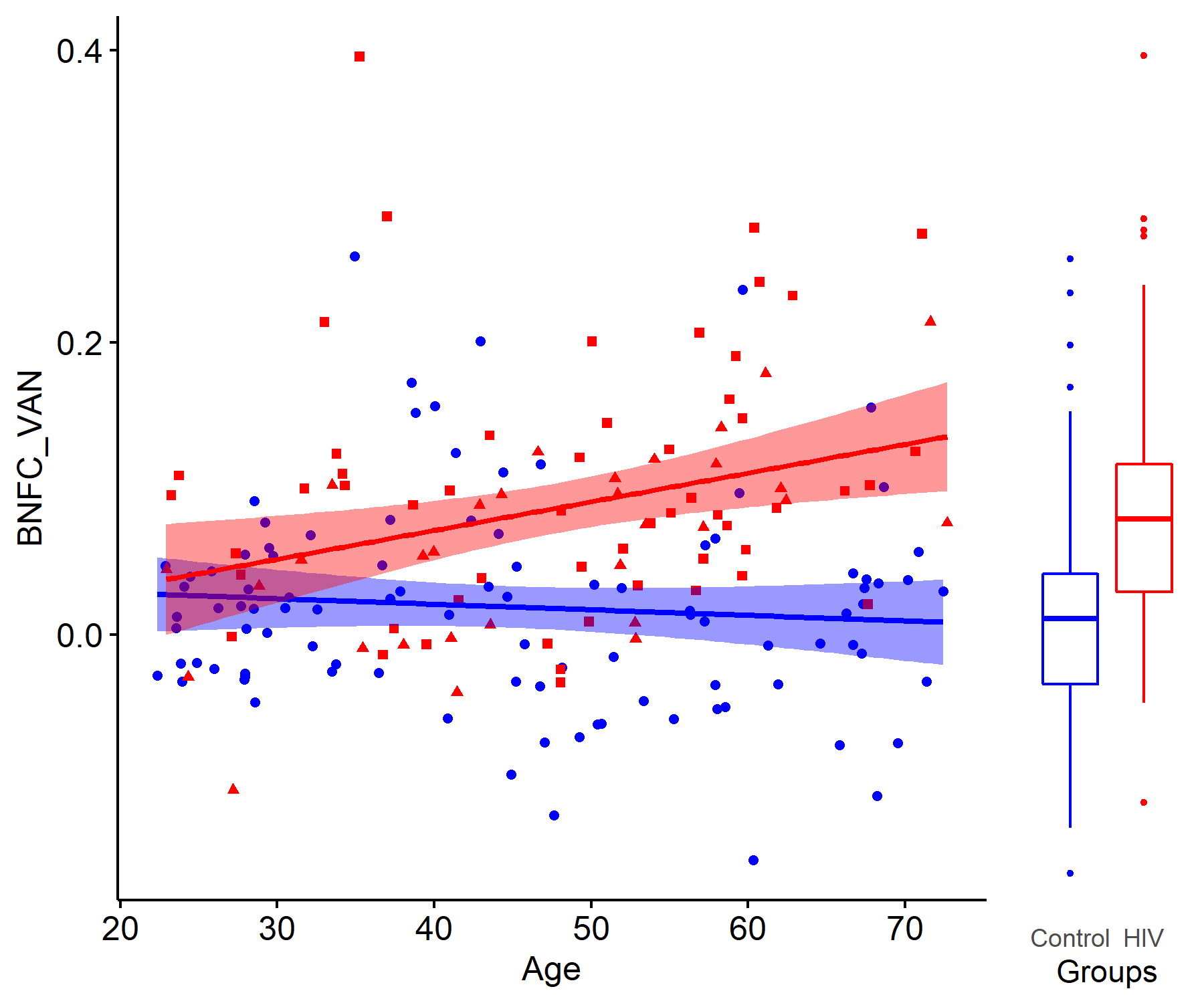

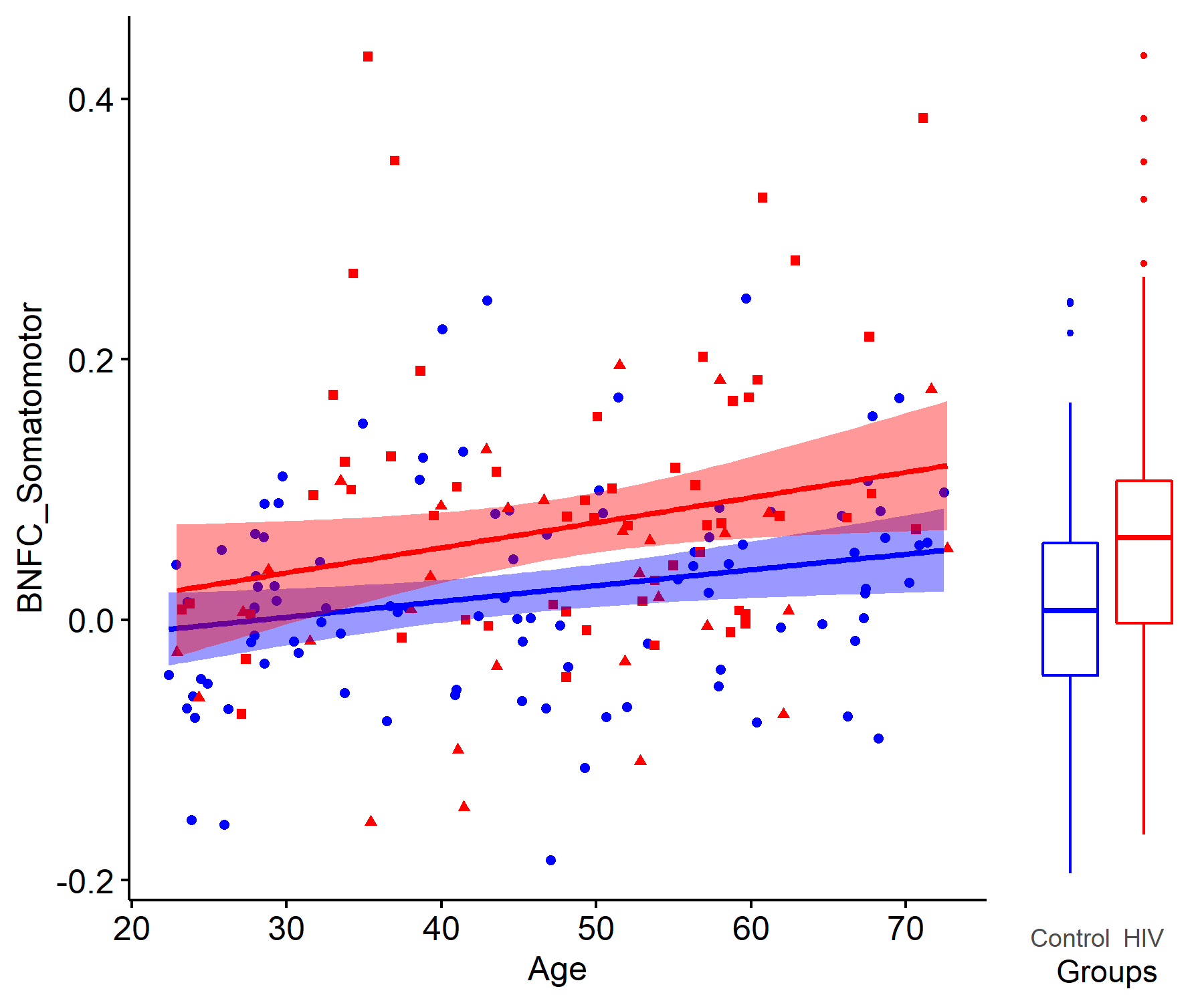

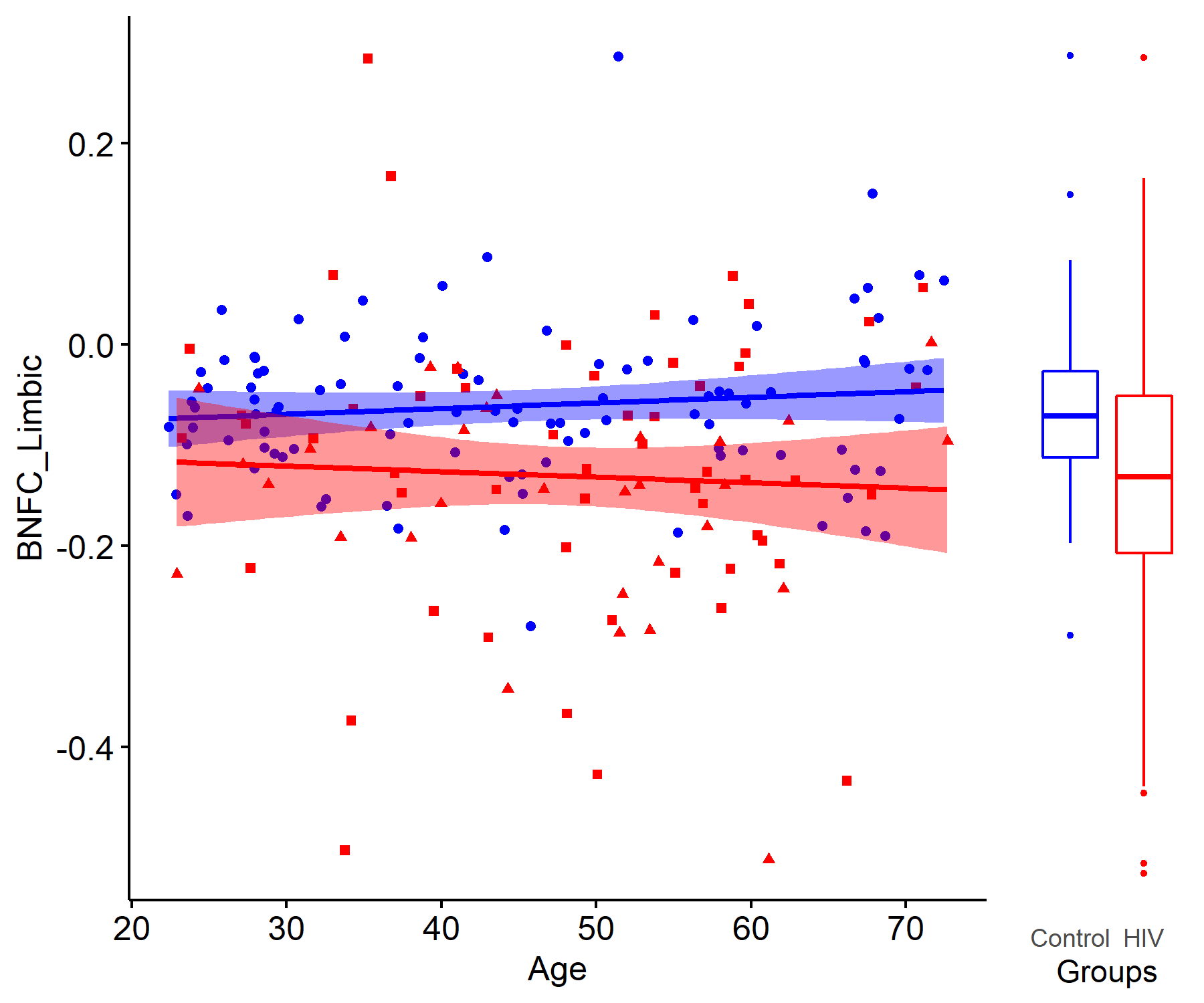

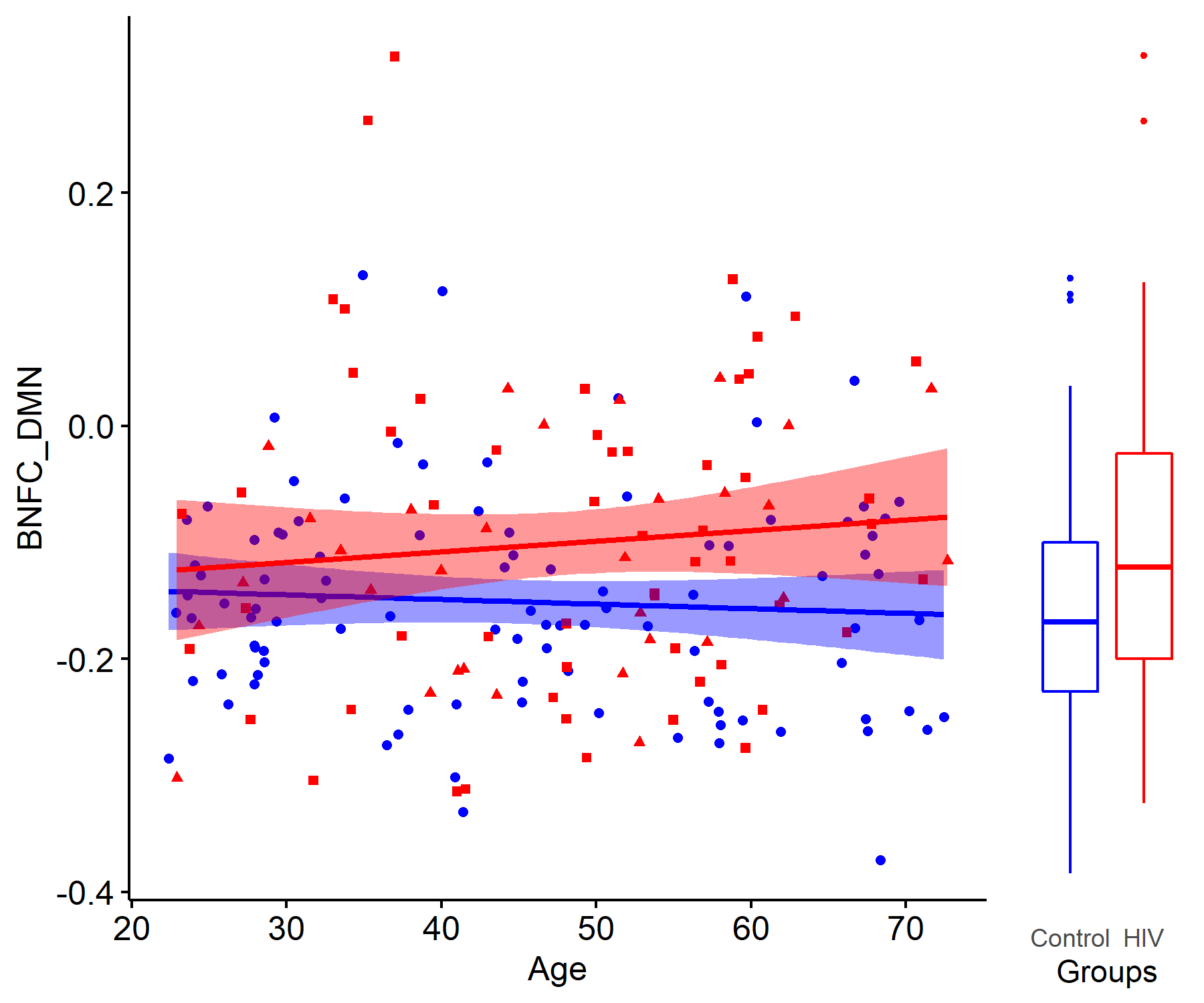

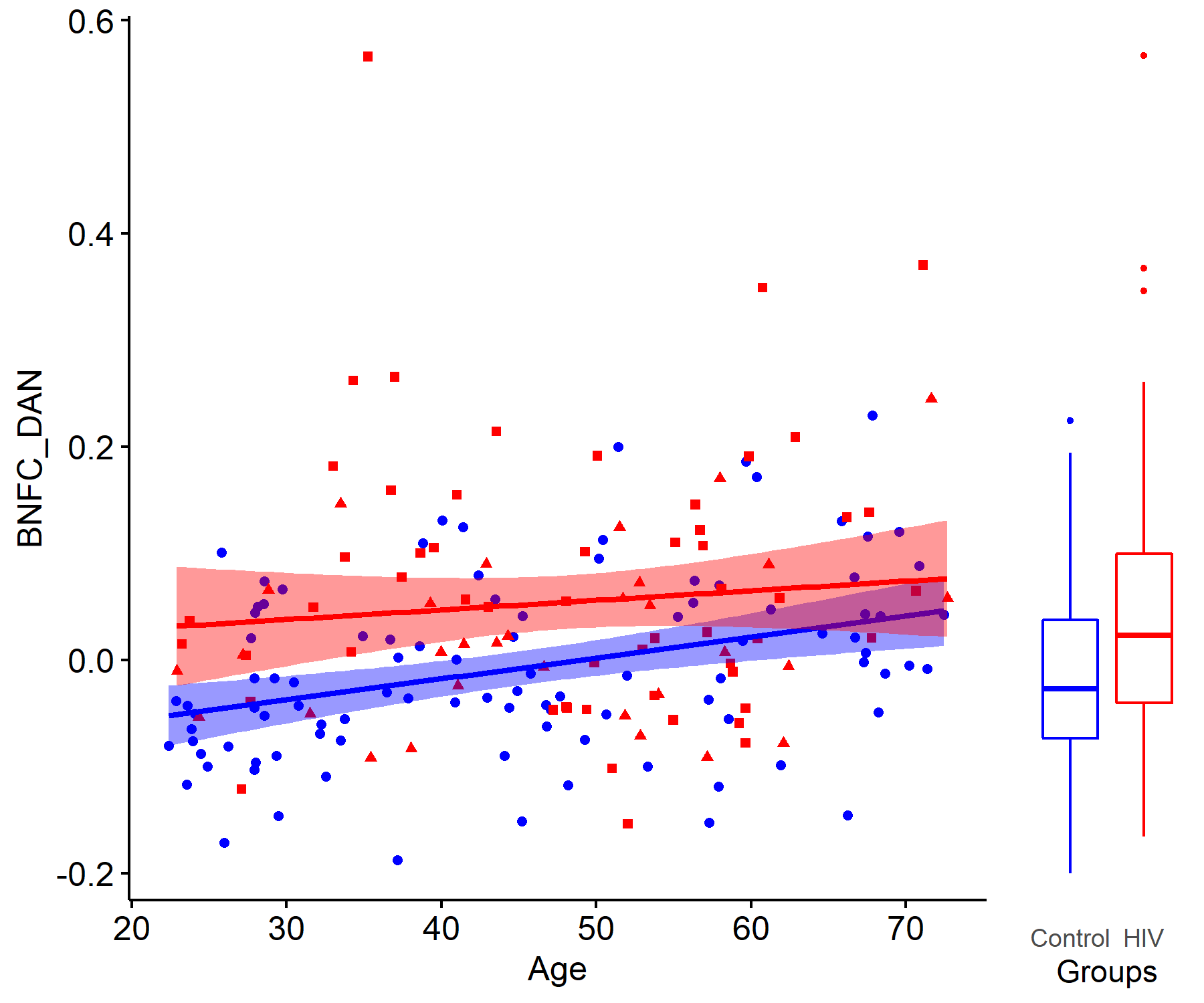

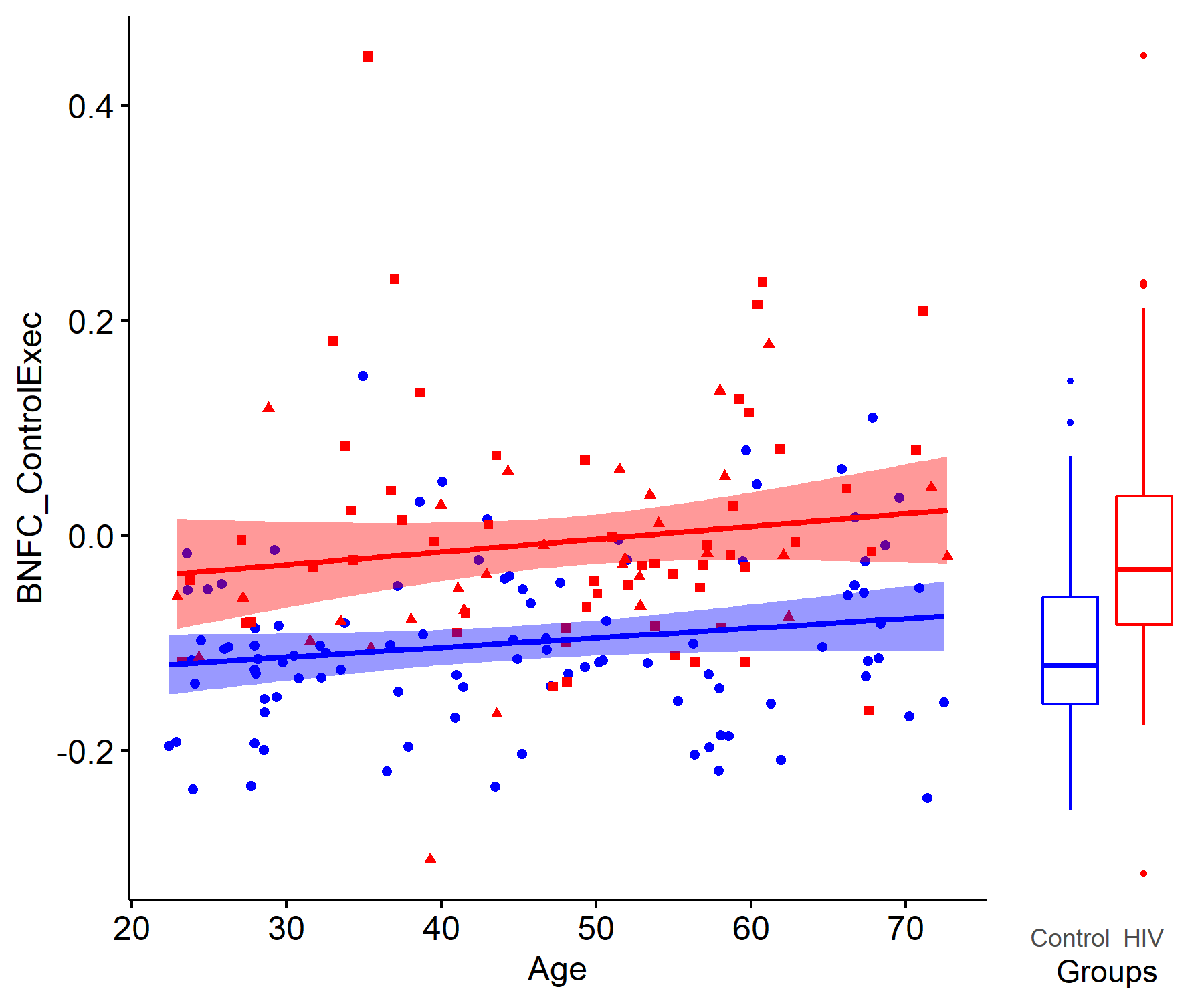

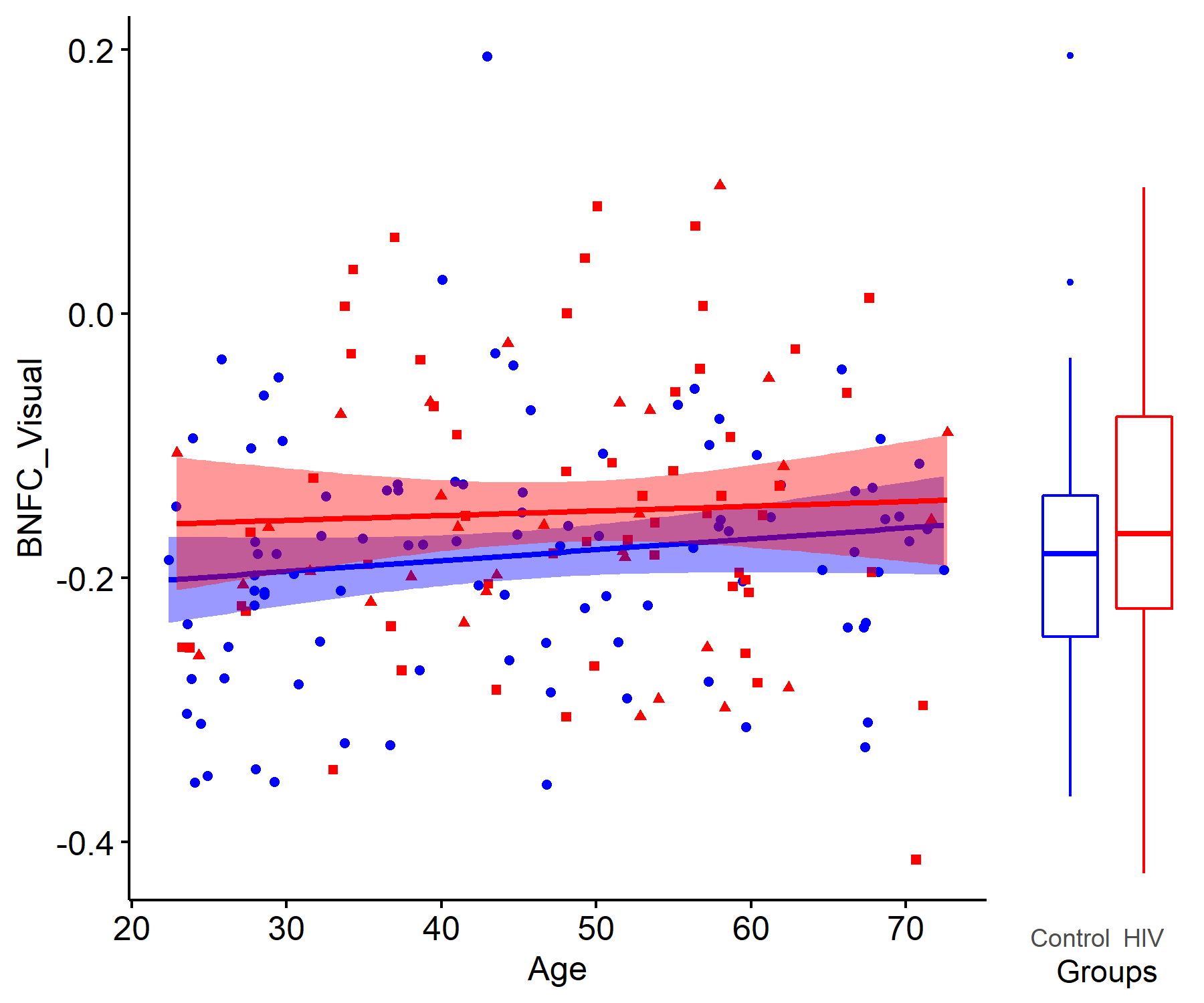


*Figure S3: Between-Network Functional Connectivity in Global Signal Regression (GSR) processed data for BNFC. Data were reprocessed using global signal regression (GSR) and BNFC was calculated as before. As expected, z values became more negative, however the overall pattern of findings remained stable in that FC increased with age, and there is added increase in PWH. One exception was the limbic network, which previous studies have identified as being unstable (Sbaihat et al. 2021, Chen et al. 2015). Scatter plots display z values of each between-network functional connectivity metric by age, with uninfected controls in blue and PWH in red. HAND status is differentiated by shape for display purposes. Linear fits for each group are displayed with 95% confidence intervals. Boxplots displaying group differences are added to the right of each plot, and visual representations of each network are inset.*


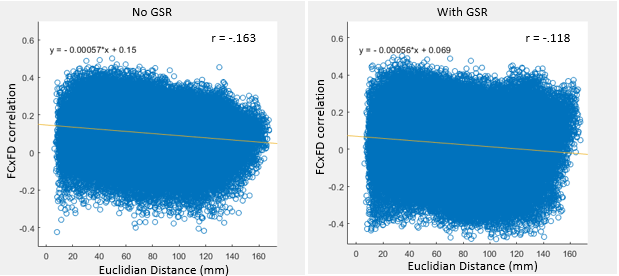


*Figure S4: Correlation vs distance plots. To examine the impact of motion on connectivity distance, functional connectivity between each pair of the 512 AAL atlas regions was correlated with participant mean framewise displacement, and the resulting correlation was plotted against each pair’s Euclidian distance. This was performed for our data both with and without global signal regression. These results replicate a commonly seen relationship between distance and motion correlation, and therefore caution is warranted when comparing long versus short range correlations (Ciric et al., 2017). However, importantly, our correlation coefficients were comparatively small and did not change drastically after global signal regression (-0.163 to -0.118). There was also negligible change in slope after global signal regression (-.00057 to -.00056).*


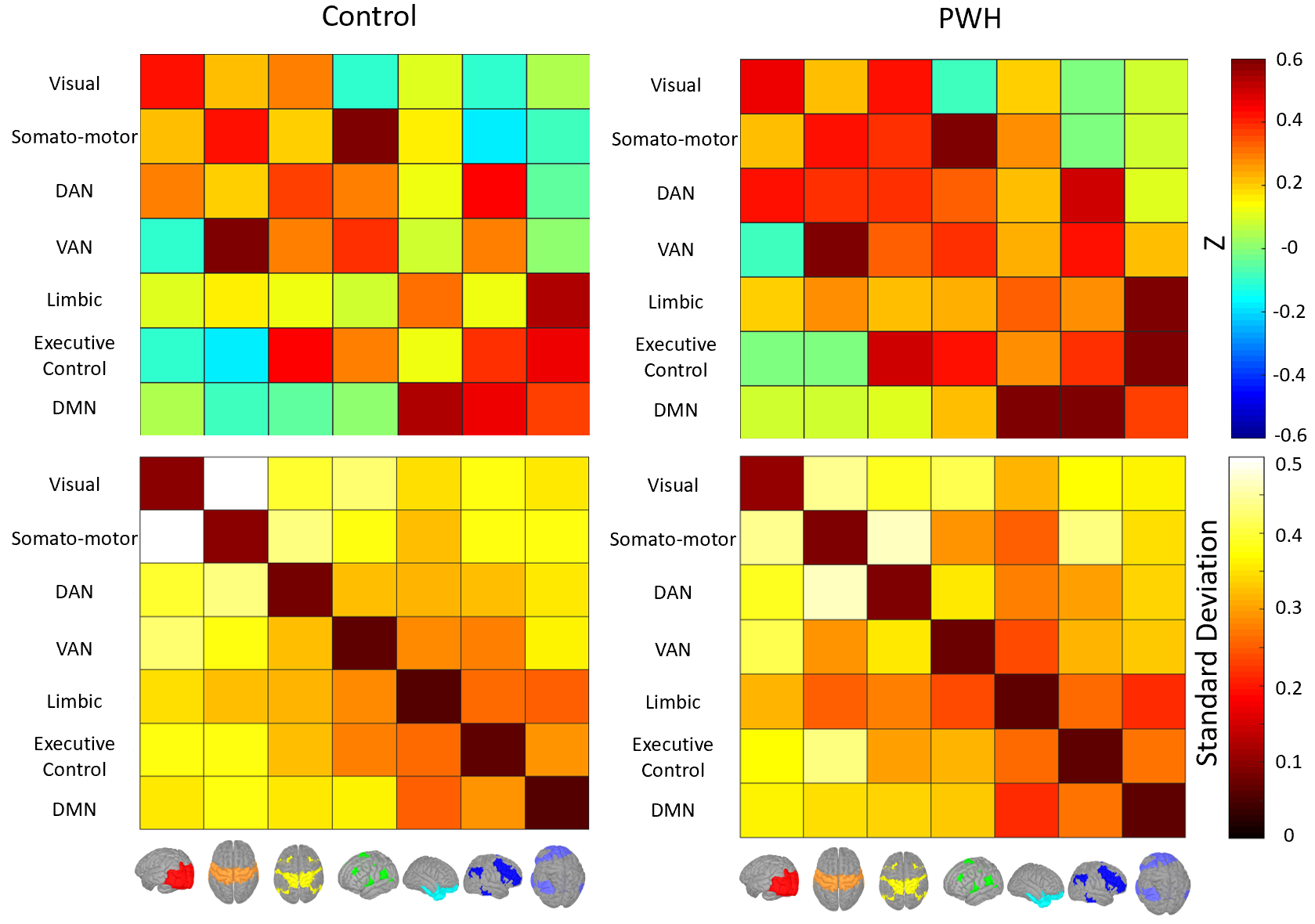


*Figure S5: Group Average (Top) and Standard Deviation (Bottom) Pairwise Functional Connectivity. Between- and within- (diagonal) network connectivity matrices averaged by groups. Matrices are symmetric with labels on the left, and visual representations of each network displayed on the bottom. Color bar to the right applies to both matrices in respective rows and represents Z values (top), with warm colors representing positive functional connectivity and cool colors representing negative functional connectivity, and standard deviation (bottom).*


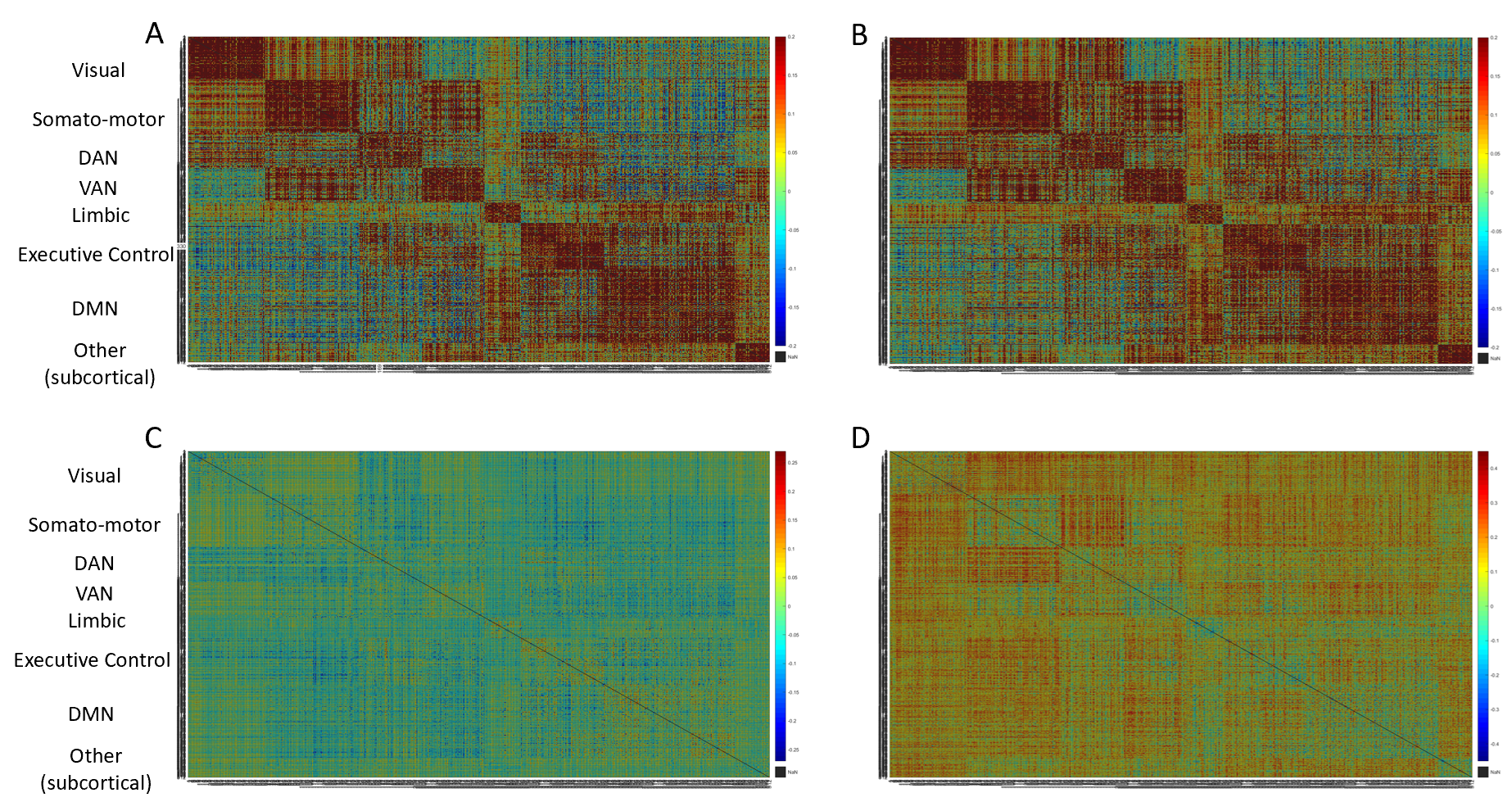


*Figure S6: Matrix Comparisons with the AAL Parcellation. To compare the 512 AAL region atlas to our original 7 network atlas, we organized the 512 regions by the 7 network parcellation. Group averages (top) for control (A) and PWH (B) are displayed. C displays the subtraction matrix, and D displays the age correlation matrix.*


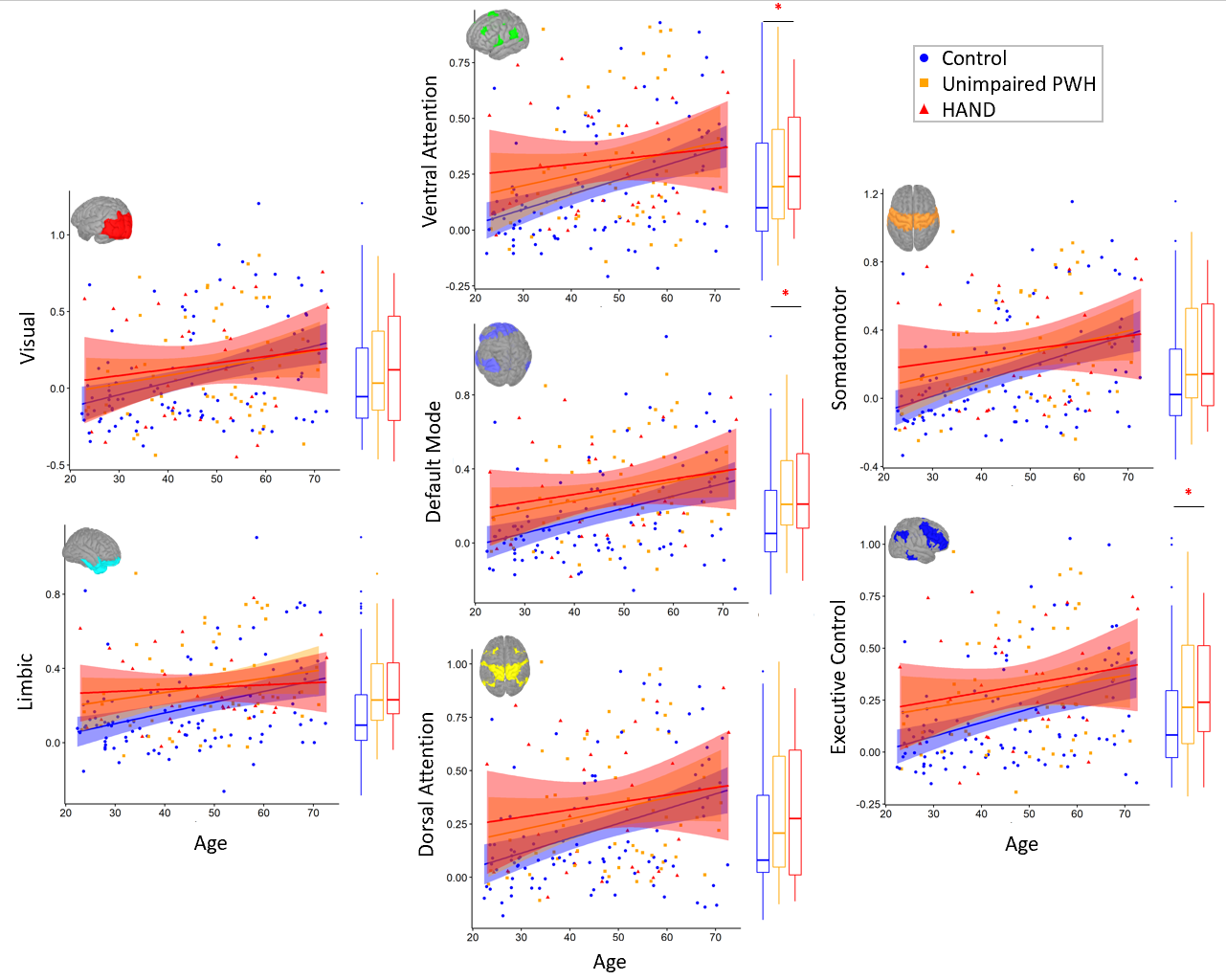


*Figure S7: Between-Network Functional Connectivity Split by HAND. The correlation between each network was calculated, z transformed, and then averaged for each network. Pairwise comparisons showed group differences in uninfected controls versus participants with HAND in VAN, DMN, and ECN (all p<.05). Scatter plots display z values of each between-network functional connectivity metric by age, with uninfected controls in blue and unimpaired PWH in yellow, and participants with HAND in red. Linear fits for each group are displayed with 95% confidence intervals. Boxplots displaying group differences are added to the right of each plot, and visual representations of each network are inset. * p<.05.*


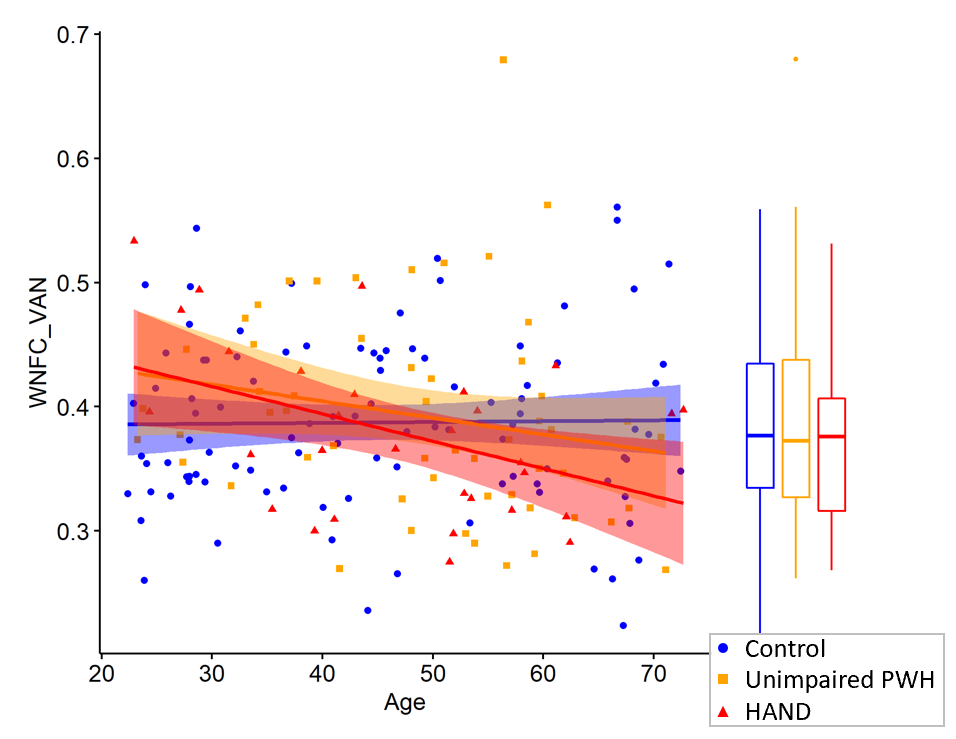


*Figure S8: Within-Network Functional Connectivity for the Ventral Attention Network. To probe the HIV by Age interaction effect and examine the interaction in the context of HAND, separate trend lines were made for unimpaired PWH and those with HAND. Pairwise examinations revealed that the control vs. HAND comparison showed a significant interaction of group by age (p = .032), while the control vs. unimpaired PWH comparison failed to display a significant group by age interaction (p=.143). This suggests that cognitive impairment may be driving this differential aging trajectory, although further study is needed, given the PWH and HAND groups did not differ in trajectory (p>.05).*
